# Wastewater Surveillance for Identifying SARS-CoV-2 Infections in Long-Term Care Facilities, Kentucky, USA, 2021–2022

**DOI:** 10.3201/eid3003.230888

**Published:** 2024-03

**Authors:** James W. Keck, Reuben Adatorwovor, Matthew Liversedge, Blazan Mijotavich, Cullen Olsson, William D. Strike, Atena Amirsoleimani, Ann Noble, Soroosh Torabi, Alexus Rockward, Mohammad Dehghan Banadaki, Ted Smith, Parker Lacy, Scott M. Berry

**Affiliations:** University of Kentucky, Lexington, Kentucky, USA (J.W. Keck, R. Adatorwovor, M. Liversedge, C. Olsson, W.D. Strike, A. Amirsoleimani, A. Noble, S. Torabi, A. Rockward, M. Dehghan Banadaki, S.M. Berry);; University of Louisville, Louisville, Kentucky, USA (T. Smith);; Trilogy Health Services, LLC, Louisville (P. Lacy)

**Keywords:** COVID-19, wastewater-based epidemiologic monitoring, SARS-CoV-2, viruses, respiratory infections, population surveillance, long-term care, viruses, United States

## Abstract

Persons living in long-term care facilities (LTCFs) were disproportionately affected by COVID-19. We used wastewater surveillance to detect SARS-CoV-2 infection in this setting by collecting and testing 24-hour composite wastewater samples 2–4 times weekly at 6 LTCFs in Kentucky, USA, during March 2021–February 2022. The LTCFs routinely tested staff and symptomatic and exposed residents for SARS-CoV-2 using rapid antigen tests. Of 780 wastewater samples analyzed, 22% (n = 173) had detectable SARS-CoV-2 RNA. The LTCFs reported 161 positive (of 16,905) SARS-CoV-2 clinical tests. The wastewater SARS-CoV-2 signal showed variable correlation with clinical test data; we observed the strongest correlations in the LTCFs with the most positive clinical tests (n = 45 and n = 58). Wastewater surveillance was 48% sensitive and 80% specific in identifying SARS-CoV-2 infections found on clinical testing, which was limited by frequency, coverage, and rapid antigen test performance.

Persons living in long-term care facilities (LTCFs) have experienced disproportionate illnesses and deaths from the COVID-19 pandemic. By June 2020, >50,000 COVID-19 deaths had occurred in LTCF residents in the United States, an estimated 43% of all US COVID-19 deaths in a group comprising <1% of the US population (*1*). Nearly 2 years later, the COVID-19 pandemic continues to cause disproportionate illnesses and deaths in this vulnerable population and is responsible for >200,000 LTCF resident deaths in the United States (*2*).

Early detection of SARS-CoV-2 infection in LTCF staff or residents is an important strategy to mitigate SARS-CoV-2 transmission. Routine symptom screening of LTCF employees and residents was the primary strategy to detect infections early in the pandemic. However, symptom screening misses persons with presymptomatic or asymptomatic SARS-CoV-2 infection (*3*) and performs similarly to the flip of a coin for identifying persons with SARS-CoV-2 infection (*4*). Clinical testing, which was heavily constrained early in the pandemic, became the preferred screening approach as testing capacity increased in 2020. Federal and state guidance encouraged routine clinical testing of unvaccinated asymptomatic LTCF staff with the frequency determined by the level of community transmission (*5*). However, routine clinical testing of large numbers of asymptomatic persons is expensive, invasive, and inefficient and may be inaccurate depending on the type of clinical test used.

Wastewater surveillance provides an alternative strategy for SARS-CoV-2 detection by evaluating samples of wastewater for the presence of viral biomarkers like RNA (*6*). Persons infected with SARS-CoV-2 shed virus in their feces (*7*); early in the pandemic, scientists reported detecting the virus in the wastewater of urban areas (*8*,*9*). Many municipalities across the United States surveilled wastewater at treatment plants for SARS-CoV-2, while universities tested wastewater at the building level; researchers used the collected data to trigger enhanced clinical testing that led to identifying persons with previously unknown SARS-CoV-2 infections (*10*,*11*). We implemented wastewater surveillance to detect SARS-CoV-2 infection at LTCFs and assessed its performance using routine clinical testing data.

## Methods

### Study Population and Site Selection

We collaborated with a LTCF organization that manages >100 LTCFs across the upper Midwest of the United States. We identified LTCF study sites on the basis of their proximity to our research laboratory in Lexington, Kentucky; their sewer system design allowing for facility-specific sampling; and presence of SARS-CoV-2 infections. We selected 3 LTCFs in Lexington and 3 LTCFs in Louisville, Kentucky; each facility served 67–160 residents and had 76–117 staff. The University of Kentucky Institutional Review Board approved this study (IRB no. 62384).

### Wastewater Collection

We collected 24-hour composite LTCF effluent wastewater samples 2–3 times/week at Louisville sites and 3–4 times/week at Lexington sites. We initiated wastewater sampling in both cities on March 19, 2021, and concluded wastewater collection on December 17, 2021, at the Louisville sites and on February 18, 2022, at the Lexington sites. We installed Teledyne ISCO GLS composite autosamplers (https://www.teledyneisco.com/water-and-wastewater/gls-compact) with 12V batteries in effluent sewer pipes via the manhole access closest to the LTCF. The autosamplers collected 100 mL of wastewater effluent every 20 minutes for 24 hours. Ice packed around the autosampler collection jug cooled the wastewater to a target temperature <4°C to minimize degradation of nucleic acids. After a 24-hour cycle of composite sampling, we transported 250 mL of the composite wastewater sample on ice to the laboratory for analysis and disposed of the remaining sample in the sewer.

Before initiating wastewater surveillance at one LTCF, we flushed RNA encoding for jellyfish-derived enhanced green fluorescent protein (eGFP) into a toilet and collected 5-minute fractionated wastewater samples to measure the durability of the RNA signal in the wastewater effluent. We used real-time PCR to measure eGFP RNA in the fractionated wastewater samples. We detected eGFP in the initial wastewater fraction collected 3 minutes after flushing and in most of the wastewater fractions (11/16) over the 2-hour collection window; those findings supported the use of a 20-minute sampling cadence.

### Quantification of SARS-CoV-2 in Wastewater

We extracted RNA from wastewater samples on the same day as sample collection. To address the heterogeneous distribution of biologic material in wastewater, we analyzed 8 replicates of 250 μL from each wastewater sample. We used exclusion-based sample preparation (ESP) to extract nucleic acids from the wastewater replicates. We previously published a detailed description of this method for analysis of SARS-CoV-2 RNA in wastewater (*12*). In brief, we lysed samples and added paramagnetic particles (PMPs) (SeraSil-Mag; Cytiva, https://www.cytivalifesciences.com). We vortexed the samples, heated them at 50°C for 20 minutes, and then tumbled them for 20 minutes. We loaded these samples into an ESP device (Extractman; Gilson, Inc., https://www.gilson.com) with wash buffers and processed the replicates as previously described. We heated the purified PMP-RNA complexes for 20 minutes at 70°C to elute the RNA. We tracked RNA extraction efficiency using negative wastewater samples spiked with known concentrations of whole SARS-CoV-2 virus (BEI Resources, https://www.beiresources.org).

We amplified and quantified ESP-purified RNA via real-time quantitative PCR using the CDC-recommended SARS-CoV-2 N1 gene primer and probe sequences (*13*). We used positive and negative controls with each PCR plate for quality assurance of the PCR process. For the positive control, we added SARS-CoV-2 RNA (BEI Resources) to the reaction. We calculated wastewater SARS-CoV-2 concentrations on the basis of quantification cycle (Cq) values and the Roche LightCycler 2nd derivative maximum algorithm (https://diagnostics.roche.com). We translated Cq values into SARS-CoV-2 genomic concentrations using a standard curve (r^2^ = 0.985) constructed from serial dilutions of the BEI positive-control RNA. We reported wastewater SARS-CoV-2 values as the arithmetic average of 8 aliquots (or the number of aliquots with valid results) from a given sample in units of genome copies per milliliter of wastewater (gc/mL).

### Clinical Testing

Clinical testing of LTCF staff and residents for SARS-CoV-2 occurred in accordance with LTCF policy and at the discretion of individual staff choosing to test outside the workplace. We received deidentified positive and negative clinical test results from staff and residents during the study period from the 6 facilities with wastewater testing. The LTCF organization used antigen-based point of care SARS-CoV-2 tests (Binax Now; Abbott, https://www.abbott.com) for routine staff screening. Employees who sought SARS-CoV-2 testing outside of their employer’s testing program were required to report their test results to the LTCF organization.

Testing frequency of staff and residents followed federal and state guidance (https://chfs.ky.gov/cv19/LTCFSurveillanceTestingFAQs.pdf). In accordance with that guidance, LTCF-based clinical testing happened routinely for unvaccinated staff working onsite, for symptomatic residents and employees, and for all residents and staff after a positive test result in a resident or staff member at the facility. Frequency of testing asymptomatic unvaccinated staff depended on the level of SARS-CoV-2 transmission in the county in which the facility was located and varied from 2 times/week (high transmission) to weekly (substantial transmission) to monthly (moderate/low transmission) according to a color-coded map (https://chfs.ky.gov/agencies/os/oig/dhc/Pages/cvltc.aspx) based on CDC transmission risk criteria (https://covid.cdc.gov/covid-data-tracker/#county-view).

### Data Analysis

We provide a descriptive summary of the wastewater RNA concentrations and clinical test data using counts, proportions, means, medians, and SDs. When a person had 2 consecutive SARS-CoV-2 positive clinical test results within 21 days of each other, we excluded the second test result from the final analytic dataset because it likely represented the same SARS-CoV-2 infection. We defined a cluster of cases when >1 LTCF resident from the same facility tested positive for SARS-CoV-2 within 14 days. To evaluate whether wastewater testing identified SARS-CoV-2 in LTCFs earlier than routine clinical screening, we conducted a lead/lag time correlational analysis. We estimated the correlation between the wastewater RNA concentration and the number of identified positive clinical SARS-CoV-2 infections at each LTCF and offset clinical testing data by 1‒7 days before and after the wastewater data collection date.

We estimated the SARS-CoV-2 wastewater contribution per known clinical case by dividing wastewater concentrations by the number of clinical cases to obtain an average wastewater viral concentration per clinical case. We used weekly averaged wastewater SARS-CoV-2 concentrations and total weekly clinical cases for this calculation to moderate differences in sampling and testing frequency between facilities. We excluded weeks when there were no clinical cases because this would result in dividing by 0.

We estimated the concentration of SARS-CoV-2 RNA in wastewater corresponding to >1 clinically confirmed case at an LTCF by fitting a negative binomial regression model to the weekly average number of positive clinical tests (Appendix). During the model fitting procedure, we used the log-link function and the total number of LTCF residents as the exposure variable.We used the incidence density ratios for positive SARS-CoV-2 test results for each LTCF to estimate the incidence rate or probability of identifying a clinical case in an LTCF during the surveillance period based on the wastewater signal. We assumed that SARS-CoV-2 RNA detected in the wastewater during the surveillance day correlated with symptomatic or asymptomatic persons infected and shedding SARS-CoV-2 virus into the wastewater. We used weekly RNA wastewater averages because of the limited number of wastewater samples collected during the week.

Last, we evaluated the sensitivity and specificity of wastewater surveillance for detecting SARS-CoV-2 infections identified through clinical testing. We categorized wastewater samples categorized as either positive or negative using various SARS-CoV-2 RNA concentration threshold values (0–250 gc/mL). In our analysis, we defined clinical test positivity as a positive clinical test result observed during the 1-week window after each wastewater measurement at that facility. We constructed 2 × 2 contingency tables to allocate positive and negative wastewater and clinical testing results and calculated the sensitivity and specificity of wastewater testing at each wastewater SARS-CoV-2 RNA concentration threshold. The primary analysis used SARS-CoV-2 infections identified in staff and residents; a secondary analysis used only resident case data because staff may not defecate at work and they isolated at home following a positive test. We used SAS version 9.4 (SAS Institute Inc., https://www.sas.com) for the statistical analyses.

## Results

**Figure 1:**
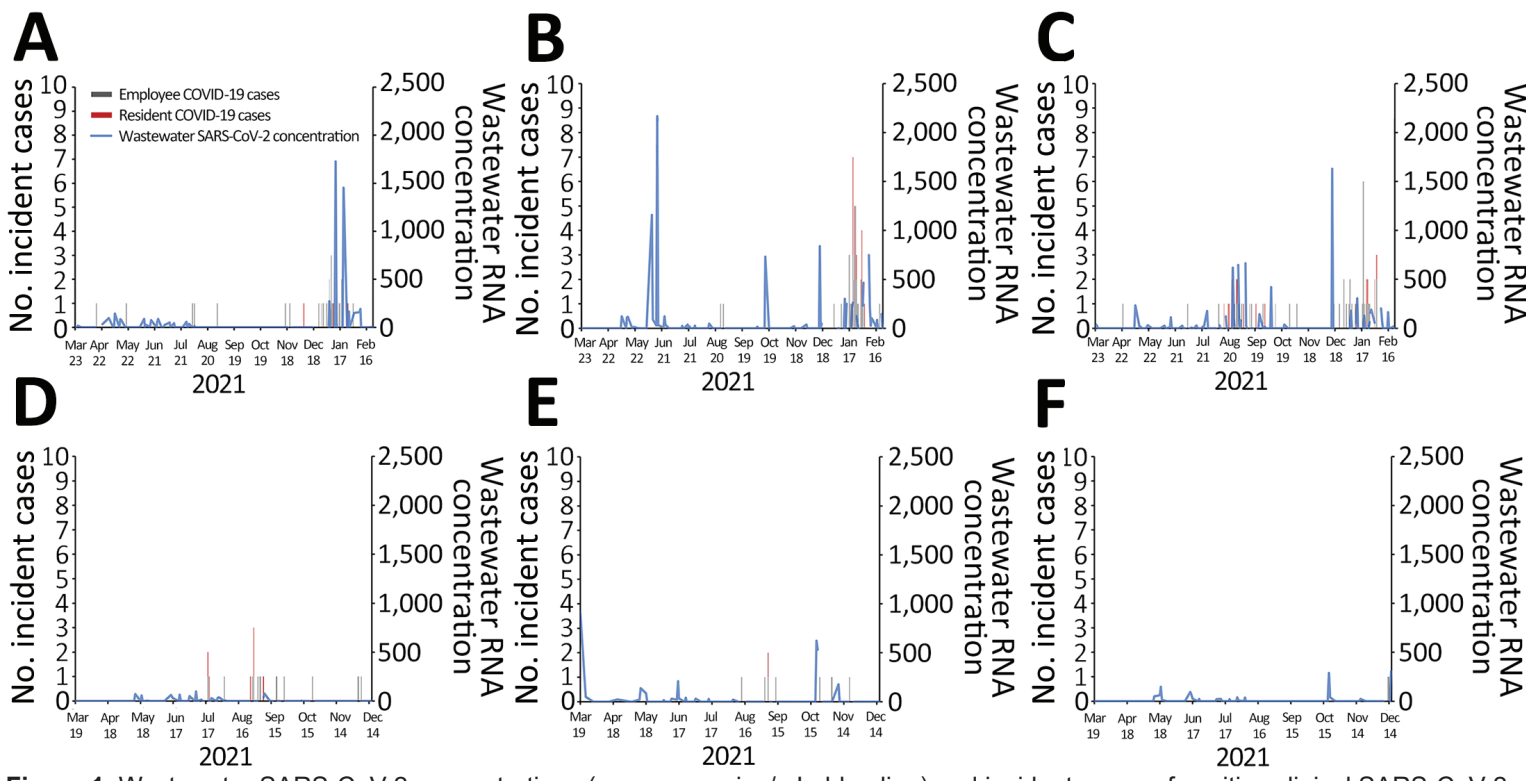
Wastewater SARS-CoV-2 concentrations (genome copies/mL; blue line) and incident cases of positive clinical SARS-CoV-2 tests (red bars for residents, gray bars for staff) from 6 long-term care facilities (A‒F), Kentucky, USA, March 2021‒February 2022.

During March 19, 2021–February 18, 2022, we collected and analyzed 780 composite wastewater samples from the 6 LTCFs (98–160 samples per facility (Table 1). An additional 31 wastewater samples were collected but not processed due to reagent shortages (n = 21), processing delays following winter storms (n = 9), or contamination during laboratory extraction (n = 1). We identified SARS-CoV-2 RNA in 18%–27% of wastewater samples at each facility at levels of 0–1,726 gc/mL. The SARS-CoV-2 wastewater signal varied over time and across facilities (Figure 1); positivity was greater during December 2022–January 2023, which also was when most of the positive SARS-CoV-2 clinical tests were reported from facilities A–C that had ongoing wastewater surveillance.

**Table 1:** Characteristics of clinical and wastewater testing for SARS-CoV-2 at 6 long-term care facilities, Kentucky, USA, 2021–2022*

Site	Person	Population†	Total no. clinical tests (% RAT†)	SARS-CoV-2 positive, no. (%)	WW surveillance duration, d	WW samples	WW SARS-CoV-2 detection, no. (%)
A	Resident	75	558 (94.8)	3 (0.5)	338	160	32 (20.0)
	Staff	89	1,607 (92.8)	24 (1.5)			
B	Resident	65	525 (94.3)	14 (2.7)	338	160	42 (26.3)
	Staff	85	1,475 (87.1)	31 (2.1)			
C	Resident	95	2,736 (99.4)	17 (0.6)	338	160	43 (26.9)
	Staff	117	3,808 (95.4)	41 (1.1)			
D	Resident	160	730 (98.9)	6 (0.8)	274	102	18 (17.6)
	Staff	106	1,965 (93.6)	12 (0.6)			
E	Resident	91	625 (96.8)	1 (0.2)	274	100	18 (18.0)
	Staff	98	1,951 (93.6)	7 (0.4)			
F	Resident	67	94 (43.6)	1 (1.1)	274	98	20 (20.4)
	Staff	76	831 (85.9)	4 (0.5)			
All	Resident	553	5,268 (97.0)	42 (0.8)	274–338	780	173 (22.2)
	Staff	571	11,637 (92.7)	119 (1.0)			
	All	1,124	16,905 (94.0)	161 (1.0)			

*RAT, rapid antigen test; WW, wastewater. †Population at time of study conclusion.

During the wastewater surveillance period, the LTCF organization reported the results of 16,905 COVID-19 tests from residents (n = 5,268) and staff (n = 11,637) at the 6 facilities (Table 1). Residents had 42 (0.8%) positive tests and staff had 119 (1.0%) positive tests. In 4 instances, >1 LTCF resident from the same facility tested positive for SARS-CoV-2 within 14 days, which we designated as a cluster of cases. Clusters included 4–14 residents and lasted 13–47 days. Wastewater positivity varied in these clusters; 25%–75% of samples had measurable SARS-CoV-2 RNA (Table 2).

**Table 2:** SARS-CoV-2 case clusters and associated wastewater signal characteristics at 4 long-term care facilities, Kentucky, USA, 2021–2022*

Characteristic	Facility			
	B	C	C	D
Case cluster				
No. residents infected	14	10	7	4
Duration, d	15	47	24	13
Wastewater signal				
Period since previous positive signal, d	6	5	6	23
Magnitude of previous signal, genome copies/mL	250.7	29.8	177.9	6.8
Signal on day of initial positive clinical test, genome copies/mL	208.1	NA	53.5	NA
Time from initial case to positive signal, d	0	2	0	12
Signal range, genome copies/mL	0–467	0–663	0–687	0–39
Fraction of samples with SARS-CoV-2 detected	6/8	12/26	8/13	1/4

*Case clusters were defined as >1 resident testing positive for SARS-CoV-2 within 14 d at the same facility. Two clusters occurred at the same facility at different time points. NA, not applicable because no wastewater sample collected that day.

**Figure 2:**
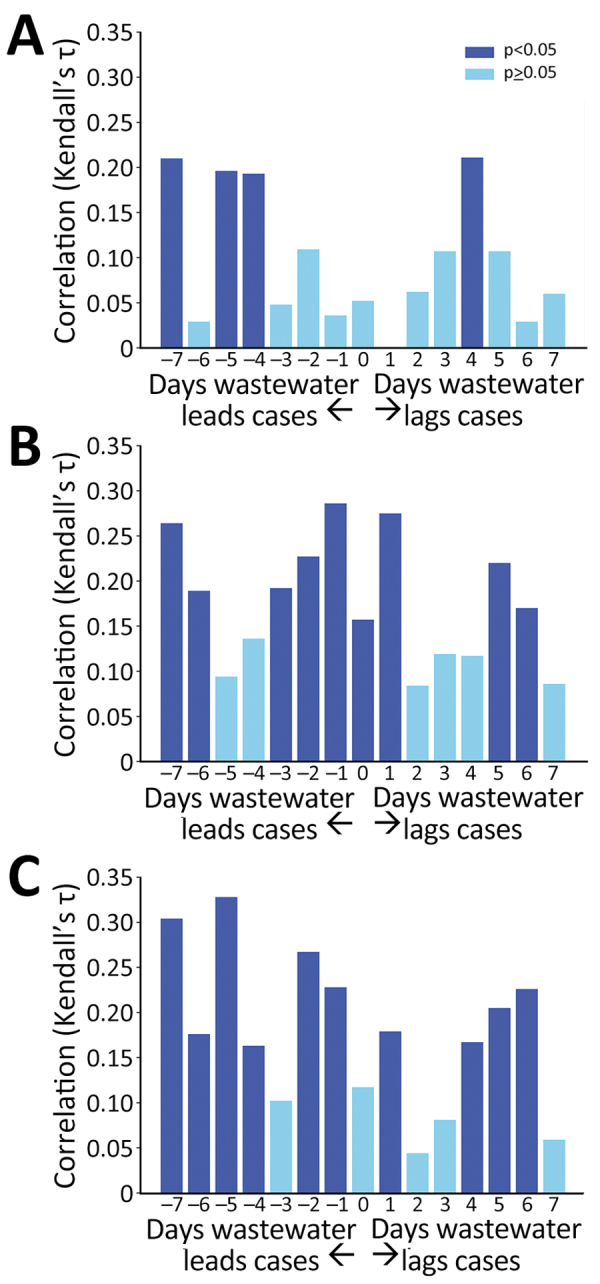
Time shifted (−7 to +7 days) correlation between wastewater SARS-CoV-2 signal and positive SARS-CoV-2 clinical tests at 3 long-term care facilities with >20 positive clinical tests (facility A = 27, facility B = 58, and facility C = 45), Kentucky, USA, March 2021‒February 2022.

The wastewater signal had a statistically significant correlation with clinical testing results. Facilities with <20 positive clinical tests showed poor correlation with the wastewater signal. However, at the 3 facilities with >20 known cases, we observed significant correlations across time shifts of the wastewater data from 7 days before to 6 days after clinical test dates (Figure 2). The strongest correlations occurred with the wastewater signal shifted 1–6 days before the clinical test dates.

**Figure 3:**
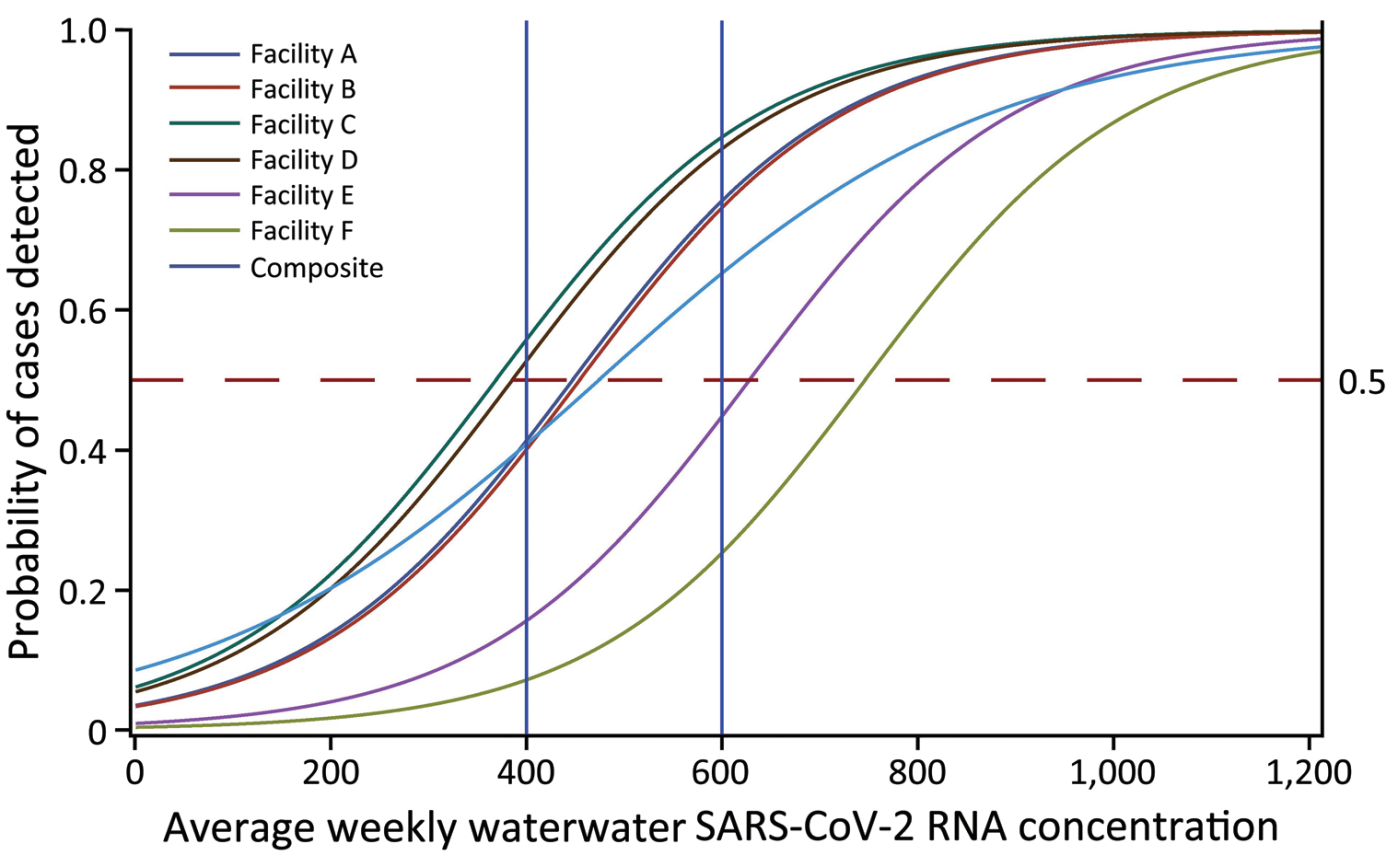
The probability of a positive SARS-CoV-2 clinical test by long-term care facility as a function of the average weekly wastewater SARS-CoV-2 concentration (genome copies/mL) at that facility, Kentucky, USA, March 2021‒February 2022. Facility-specific curves are A–F; the final curve is a composite curve that uses data from all 6 facilities. Vertical blue lines at 400 and 600 gc/mL serve as reference points to identify site-specific wastewater signal thresholds when the probably of detecting a SARS-CoV-2 case is >0.5.

On average, each identified clinical case corresponded to a wastewater concentration of 26.9 gc/mL. Using a log-linear incidence density model, we estimated the wastewater concentration associated with a probability of >0.5 clinically confirmed cases to 206–743 gc/mL (Figure 3); the estimate at the 3 facilities with the largest number of clinically confirmed cases was 206–336 gc/mL.

**Figure 4:**
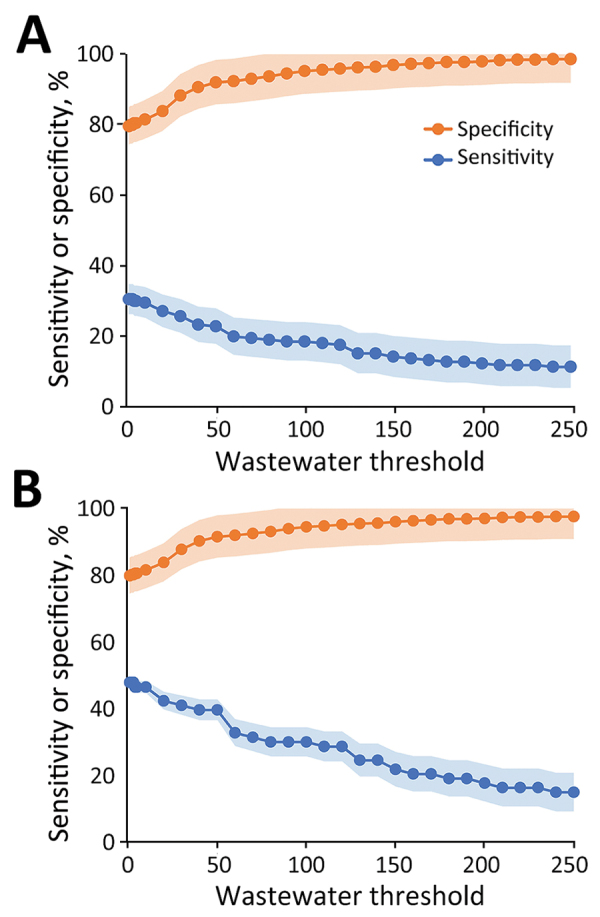
Sensitivity and specificity of SARS-CoV-2 wastewater surveillance for identifying positive SARS-CoV-2 clinical tests as a function of the wastewater SARS-CoV-2 signal strength in 6 long-term care facilities, Kentucky, USA, March 2021‒February 2022. A) Staff and residents; B) residents only. Shaded areas indicate 95% CIs.

A positive wastewater SARS-CoV-2 signal (>0 gc/mL) was 30.6% (95% CI 24.4%–36.9%) sensitive and 79.7% (95% CI 76.4%–82.9%) specific in identifying a positive clinical test result when we included test data from staff and residents (Figure 4). Wastewater sensitivity improved to 48.0% (95% CI 36.5%–59.4%) and specificity to 79.9% (95% CI 77.0%–82.9%) when we considered only clinical test data from residents. Higher wastewater signal thresholds resulted in lower sensitivity and higher specificity. A wastewater signal threshold of 30 gc/mL resulted in a sensitivity of 39.7% (95% CI 28.5%–51.0%) and specificity of 92% (95% CI 89.5%–93.6%) for identifying a LTCF resident with a positive SARS-CoV-2 test.

## Discussion

We collected and analyzed >700 wastewater samples for SARS-CoV-2 from 6 LTCFs during the second year of the COVID-19 pandemic. By pairing the wastewater data with clinical testing results from staff and residents at the 6 facilities, we evaluated the performance of wastewater surveillance for detecting clinical SARS-CoV-2 cases in this vulnerable population. Wastewater surveillance demonstrated statistically significant correlations with clinical test results, and the estimated correlation was stronger when considering the wastewater signal as leading clinical case identification; those findings suggest its potential as an early warning indicator of infection in a facility. Wastewater surveillance performance in discriminating the presence of a positive clinical SARS-CoV-2 test varied depending on the wastewater signal threshold selected and it demonstrated better specificity than sensitivity. 

Several factors affected the performance of LTCF wastewater surveillance and challenged the interpretation of the wastewater data. The population that contributed to the wastewater at an LTCF was dynamic and difficult to track. Residents were admitted and discharged, staff turnover was frequent, staff worked across multiple facilities, residents were visited by family members and friends, and visitors passed through the facilities. The frequency with which staff, visitors, and residents contributed waste to the LTCF sewer system was not known. In addition, the sewer access at facility B was where the facility’s effluent sewage joined the sewage from an adjacent apartment complex. Facility B wastewater samples may have inadvertently included wastewater from persons living in or visiting the apartment complex, which is the likely reason for the high SARS-CoV-2 RNA concentrations measured in June 2021 in the absence of identified SARS-CoV-2 infections at the facility (*14*).

Negative wastewater samples observed at facilities with known SARS-CoV-2‒infected residents could be attributed to residents wearing adult briefs secondary to fecal incontinence. For example, during a cluster of 10 resident SARS-CoV-2 infections over 13 weeks (Table 2), 3 of the residents were completely incontinent and wore adult briefs. The feces from those residents were disposed in biomedical waste receptacles rather than in the sewer system. Three other residents were partially incontinent. Feces from those residents also may not have entered the sewer system. Diversion of LTCF resident waste may reduce the sensitivity of wastewater surveillance in this setting.

Another likely cause of a negative wastewater signal in the presence of known infections is the variability with which SARS-CoV-2‒infected persons shed virus in their feces. Studies done early in the pandemic detected virus in stool samples of 29%–59% of persons with COVID-19 (*15*–*17*). The patients in those studies were hospitalized and presumably infected with nonvariant SARS-CoV-2 virus. Shedding frequency may differ in persons with milder or asymptomatic illness, of different ages, or infected with SARS-CoV-2 variants. In addition, it is unknown how vaccination status and previous SARS-CoV-2 infection affect fecal shedding. Viral shedding frequency, intensity, and duration may have outsized effects on building-level wastewater surveillance because of the small numbers of persons contributing to the wastewater.

To optimize our ability to detect SARS-CoV-2 in LTCF wastewater, we collected 24-hour composite samples using a 20-minute sampling cadence. As described in the Methods section, the results of our spiking experiment suggested that a 20-minute sampling cadence would capture RNA associated with a bowel movement flushed into the sewer system at an LTCF. Our sample collection schedule meant that we obtained wastewater samples from 37% of days in Louisville and 47% of days in Lexington during our surveillance period. Because an infected person shedding virus is likely to do so for many days, a sampling frequency of 3–4 days per week should detect the case-patients who shed virus into a facility’s wastewater system if they remain onsite during the duration of their illness.

Two additional properties of the wastewater samples may have affected our results. First, there were likely inhibitors (i.e., factors that degrade RNA, reduce PCR efficiency, or both) in the wastewater of the LTFCs because of laundry, kitchen, and janitorial activities. Detergents decreased the detectable signal of extracted RNA by ≈100-fold in 1 study (*18*), and detergents used by LTCF staff may have degraded RNA in the sewer system. We did not assess for the presence of specific inhibiting compounds and do not know how substantial their burden and effects were on our laboratory analyses. Second, wastewater is a highly heterogeneous matrix, and although we made reasonable efforts to homogenize wastewater samples (collecting composite samples, mixing composite sample before aliquoting sample for laboratory analysis, mixing laboratory sample before aliquoting for replicate analysis), variation in RT-PCR results across the 8 replicates from each composite sample suggests a heterogeneous distribution of SARS-CoV-2 virus within wastewater. Strike et al. demonstrated that our laboratory method combined with 8 replicates reliably detected SARS-CoV-2 RNA concentrations down to 100 gc/mL, and lower concentrations were observed after averaging zero and nonzero datapoints (*12*). In our study, many samples contained a mixture of positive and negative replicates. In those cases, positive replicates were averaged together with negative measurements (e.g., 0 gc/mL), often yielding average values <100 gc/mL.

We evaluated the performance of wastewater surveillance against the results of intermittent and incomplete clinical testing of LTCF staff and residents. Our LTCF partner implemented clinical testing strategies that aligned with state and federal COVID-19 guidance, which yielded pragmatic clinical testing data. Two limitations of the clinical testing protocols may have affected data quality and completeness. First, asymptomatic LTCF residents were not routinely tested; testing occurred when the resident had a known or suspected contact with a case-patient, such as a facility staff member who had tested positive. Similarly, vaccinated staff were not routinely screened. Untested but infected asymptomatic residents or vaccinated staff or a visitor to the facility may have caused a positive wastewater signal that was interpreted as a false positive, given the absence of known cases at the facility. This scenario would decrease the estimated specificity of wastewater surveillance. The second limitation was the LTCF organization’s use of rapid antigen-based SARS-CoV-2 tests for screening staff and residents. The poor sensitivity of antigen-based tests, particularly in asymptomatic persons (58% by a Cochrane meta-analysis [*19*]), likely resulted in some false-negative clinical screening tests, which would decrease the estimated specificity of wastewater surveillance.

Our study adds to the sparse literature on SARS-CoV-2 wastewater surveillance at LTCFs. A team in Italy surveilled wastewater from 5 LTCFs for several months at the end of 2020 and intermittently detected SARS-CoV-2 RNA in the wastewater of 4 of the facilities (*20*). As in our study, the presence of residents with identified COVID-19 infection only intermittently resulted in a positive wastewater signal. Researchers in Spain consistently detected SARS-CoV-2 in the wastewater effluent from an elderly residence when there were known clinical cases in the building; however, the number of known cases in a week was typically >10 (*21*). An alternative environmental surveillance approach in Canada using analysis of floor swab samples for SARS-CoV-2 demonstrated good discriminatory ability to identify COVID-19 outbreaks LTCFs (*22*).

In summary, we found that wastewater surveillance for SARS-CoV-2 performed moderately well when compared with clinical testing. Our correlational analysis indicated that a SARS-CoV-2 wastewater signal may precede the identification of clinical cases at LTCFs, which suggests that such testing could provide an early warning to trigger enhanced clinical testing or infection prevention activities, such as physical distancing. Optimizing wastewater collection and analysis methods may improve surveillance performance; however, viral and contextual factors such as fecal shedding rates, PCR inhibitors in the LTCF wastewater, and use of adult briefs likely limit wastewater surveillance performance in this setting. Improved understanding of the many potential contributors to wastewater signal variability will enhance the interpretation of this emerging surveillance strategy, which can augment traditional infection detection and prevention activities in vulnerable LTCF populations.

Appendix Additional information about wastewater surveillance for identifying SARS-CoV-2 infections in long-term care facilities, Kentucky, USA, 2021–2022.

## References

[1] Kamp J, Mathews AW. As U.S. nursing-home deaths reach 50,000, states ease lockdowns. The Wall Street Journal. 2020 Jun 16 [cited 2022 May 3]. https://www.wsj.com/articles/coronavirus-deaths-in-u-s-nursing-long-term-care-facilities-top-50-000-11592306919

[2] Chidambaram P. Over 200,000 residents and staff in long-term care facilities have died from COVID-19. Kaiser Family Foundation; 2022 Feb 3 [cited 2022 May 3]. https://www.kff.org/policy-watch/over-200000-residents-and-staff-in-long-term-care-facilities-have-died-from-covid-19

[3] Arons MM, Hatfield KM, Reddy SC, Kimball A, James A, Jacobs JR, et al.; Public Health–Seattle and King County and CDC COVID-19 Investigation Team. Presymptomatic SARS-CoV-2 infections and transmission in a skilled nursing facility. N Engl J Med. 2020;382:2081–90. 10.1056/NEJMoa2008457 32329971 PMC7200056

[4] Keck JW, Bush M, Razick R, Mohammadie S, Musalia J, Hamm J. Performance of formal smell testing and symptom screening for identifying SARS-CoV-2 infection. PLoS One. 2022;17:e0266912. 10.1371/journal.pone.0266912 35413084 PMC9004758

[5] Centers for Disease Control and Prevention. Interim infection prevention and control recommendations for healthcare personnel during the coronavirus disease 2019 (COVID-19) pandemic. 2019 [cited 2022 Oct 9]. https://www.cdc.gov/coronavirus/2019-ncov/hcp/infection-control-recommendations.html

[6] Orive G, Lertxundi U, Barcelo D. Early SARS-CoV-2 outbreak detection by sewage-based epidemiology. Sci Total Environ. 2020;732:139298. 10.1016/j.scitotenv.2020.139298 32417555 PMC7207139

[7] Chen Y, Chen L, Deng Q, Zhang G, Wu K, Ni L, et al. The presence of SARS-CoV-2 RNA in the feces of COVID-19 patients. J Med Virol. 2020;92:833–40. 10.1002/jmv.25825 32243607

[8] Wurtzer S, Marechal V, Mouchel JM, Maday Y, Teyssou R, Richard E, et al. Evaluation of lockdown effect on SARS-CoV-2 dynamics through viral genome quantification in waste water, Greater Paris, France, 5 March to 23 April 2020. Euro Surveill. 2020;25:2000776. 10.2807/1560-7917.ES.2020.25.50.2000776 33334397 PMC7812418

[9] Ahmed W, Angel N, Edson J, Bibby K, Bivins A, O’Brien JW, et al. First confirmed detection of SARS-CoV-2 in untreated wastewater in Australia: A proof of concept for the wastewater surveillance of COVID-19 in the community. Sci Total Environ. 2020;728:138764. 10.1016/j.scitotenv.2020.138764 32387778 PMC7165106

[10] Betancourt WQ, Schmitz BW, Innes GK, Prasek SM, Pogreba Brown KM, Stark ER, et al. COVID-19 containment on a college campus via wastewater-based epidemiology, targeted clinical testing and an intervention. Sci Total Environ. 2021;779:146408. 10.1016/j.scitotenv.2021.146408 33743467 PMC7954642

[11] Harris-Lovett S, Nelson KL, Beamer P, Bischel HN, Bivins A, Bruder A, et al. Wastewater surveillance for SARS-CoV-2 on college campuses: initial efforts, lessons learned and research needs. Int J Environ Res Public Health. 2021;18:4455. 10.3390/ijerph18094455 33922263 PMC8122720

[12] Strike W, Amirsoleimani A, Olaleye A, Noble A, Lewis K, Faulkner L, et al. Development and validation of a simplified method for analysis of SARS-CoV-2 RNA in university dormitories. ACS ES T Water. 2022;2:1984–91. 10.1021/acsestwater.2c00044 37552725 PMC9115885

[13] Centers for Disease Control and Prevention. CDC’s influenza SARS-CoV-2 multiplex assay. [cited 2020 Sep 6]. https://archive.cdc.gov/www_cdc_gov/coronavirus/2019-ncov/lab/multiplex.html

[14] Keck JW, Lindner J, Liversedge M, Mijatovic B, Olsson C, Strike W, et al. Wastewater surveillance for SARS-CoV-2 at long-term care facilities: mixed methods evaluation. JMIR Public Health Surveill. 2023;9:e44657. 10.2196/44657 37643001 PMC10467632

[15] Wu Y, Guo C, Tang L, Hong Z, Zhou J, Dong X, et al. Prolonged presence of SARS-CoV-2 viral RNA in faecal samples. Lancet Gastroenterol Hepatol. 2020;5:434–5. 10.1016/S2468-1253(20)30083-2 32199469 PMC7158584

[16] Zheng S, Fan J, Yu F, Feng B, Lou B, Zou Q, et al. Viral load dynamics and disease severity in patients infected with SARS-CoV-2 in Zhejiang province, China, January-March 2020: retrospective cohort study. BMJ. 2020;369:m1443. 10.1136/bmj.m1443 32317267 PMC7190077

[17] Wang W, Xu Y, Gao R, Lu R, Han K, Wu G, et al. Detection of SARS-CoV-2 in different types of clinical specimens. JAMA. 2020;323:1843–4. 10.1001/jama.2020.3786 32159775 PMC7066521

[18] Robinson CA, Hsieh HY, Hsu SY, Wang Y, Salcedo BT, Belenchia A, et al. Defining biological and biophysical properties of SARS-CoV-2 genetic material in wastewater. Sci Total Environ. 2022;807:150786. 10.1016/j.scitotenv.2021.150786 34619200 PMC8490134

[19] Dinnes J, Deeks JJ, Berhane S, Taylor M, Adriano A, Davenport C, et al.; Cochrane COVID-19 Diagnostic Test Accuracy Group. Rapid, point-of-care antigen and molecular-based tests for diagnosis of SARS-CoV-2 infection. Cochrane Database Syst Rev. 2021;3:CD013705. 33760236 10.1002/14651858.CD013705.pub2 PMC8078597

[20] Davó L, Seguí R, Botija P, Beltrán MJ, Albert E, Torres I, et al. Early detection of SARS-CoV-2 infection cases or outbreaks at nursing homes by targeted wastewater tracking. Clin Microbiol Infect. 2021;27:1061–3. 10.1016/j.cmi.2021.02.003 33601008 PMC7882920

[21] Pico-Tomàs A, Mejías-Molina C, Zammit I, Rusiñol M, Bofill-Mas S, Borrego CM, et al. Surveillance of SARS-CoV-2 in sewage from buildings housing residents with different vulnerability levels. Sci Total Environ. 2023;872:162116. 10.1016/j.scitotenv.2023.162116 36773920 PMC9911146

[22] Fralick M, Nott C, Moggridge J, Castellani L, Raudanskis R, Guttman DS, et al. Detection of COVID-19 outbreaks using built environment testing for SARS-CoV-2. NEJM Evid. 2023;2. 10.1056/EVIDoa2200203 38320044

