# Supplementary material for: Wastewater Surveillance for Identifying SARS-CoV-2 Infections in Long-Term Care Facilities, Kentucky, USA, 2021–2022
Source: Emerg Infect Dis. 2024 Mar;30(3):530–8. doi: 10.3201/eid3003.230888 (PMC10902530; doi:10.3201/eid3003.230888)
Supplement: Appendix — Additional information about wastewater surveillance for identifying SARS-CoV-2 infections in long-term care facilities, Kentucky, USA, 2021–2022. [file 23-0888-Techapp-s1.pdf]

Article DOI: http://doi.org/10.3201/eid3003.230888

*EID cannot ensure accessibility for supplementary materials supplied by authors. Readers who have difficulty accessing supplementary content should contact the authors for assistance.*

# Wastewater Surveillance for Identifying SARS-CoV-2 Infections in Long-Term Care Facilities, Kentucky, USA, 2021–2022

## Appendix

The form of the generalized linear model used to estimate clinical SARS-CoV-2 cases from wastewater data is:

$$\log n_w = \beta_0 + \beta_1 x_{w1} + \cdots + \beta_{wk} x_{wk} + logN_w$$

where $\boldsymbol{n_w}$ is the number of clinical cases in each nursing home in a week, $\boldsymbol{\beta}$ is the vector of coefficients corresponding to the parameters of the model, $\boldsymbol{x}$ is the vector of covariates to be included in the model (LTCF site and wastewater SARS-CoV-2 RNA concentration), **N** is the total exposure group, ***w*** is the week, and ***k*** represents the levels of covariates. The term $logN_w$ is the offset.

The fully expanded model is:

$$\log n_w = \alpha + \beta_1 WW + \beta_2 Site_A + \beta_3 Site_B + \beta_4 Site_D + \ \beta_5 Site_E + \beta_6 Site_F + logN_w.$$

Note that $\alpha$ is the intercept and represents the log of the number of SARS-CoV-2 clinical cases at $Site_C$.

The model's site-specific parameter estimates appear in Appendix Table 1 and overall estimates in Appendix Table 2.

The probability of detecting a SARS-CoV-2 clinical case at a specific site and wastewater SARS-CoV-2 RNA concentration is estimated by:

$$\Pr(\text{clinical case}) = \frac{\exp\{\alpha + \beta \cdot WW + \gamma \cdot Site\}}{1 + \exp\{\alpha + \beta \cdot WW + \gamma \cdot Site\}}$$

$$= \frac{\exp\{\alpha + \beta_1 WW + \beta_2 Site_A + \beta_3 Site_B + \beta_4 Site_C + \ \beta_5 Site_D + \beta_6 Site_E + logN_w)}{1 + \exp\{\alpha + \beta_1 WW + \beta_2 Site_A + \beta_3 Site_B + \beta_4 Site_C + \ \beta_5 Site_D + \beta_6 Site_E + logN_w)}$$

For a specific site, the parameter estimates from the other sites are set to zero.

**Appendix Table 1.** Site-specific parameter estimates from the wastewater SARS-CoV-2 model*

| Parameter | Estimate (SE) | Chi-Square | Pr > ChiSq |
|---|---|---|---|
| Intercept* | -2.2098 (0.5005) | 19.50 | <.0001 |
| Wastewater SARS AVG | 0.0003 (0.0011) | 0.05 | 0.8161 |
| A | -0.9074 (0.8142) | 1.24 | 0.2651 |
| B | 0.6927 (0.5847) | 1.40 | 0.2361 |
| D | -2.1982 (1.4148) | 2.41 | 0.1202 |
| E | -25.9638 (168716.7) | 0.00 | 0.9999 |
| F | -24.3478 (113948.8) | 0.00 | 0.9998 |
| Dispersion | 3.8401 (0.8694) | | |

*The intercept is the reference site and represents the log of the effect of site C, based on the reference cell coding for the model. The negative binomial dispersion parameter was estimated by maximum likelihood.

**Appendix Table 2.** Parameter estimates from the wastewater SARS-CoV-2 model

| Parameter | Estimate (SE) | Chi-Square | Pr > ChiSq |
|---|---|---|---|
| Intercept | -0.0996 (0.1941) | 0.26 | 0.6078 |
| Wastewater SARS AVG | 0.0001 (0.0006) | 0.06 | 0.8084 |
| Dispersion | 0.3092 (0.3326) | | |
